# Self-Supervised Representation Learning for Data-Efficient DRIL Classification in OCT Images

**DOI:** 10.3390/diagnostics15243221

**Published:** 2025-12-16

**Authors:** Pavithra Kodiyalbail Chakrapani, Akshat Tulsani, Preetham Kumar, Geetha Maiya, Sulatha Venkataraya Bhandary, Steven Fernandes

**Affiliations:** 1 Manipal Institute of Technology, Manipal Academy of Higher Education, Manipal 576104, India; pavithraforu@gmail.com (P.K.C.); geetha.maiya@manipal.edu (G.M.); 2 Department of Computer Science, Courant Institute of Mathematical Sciences, New York University, New York, NY 10012, USA; a97tulsani@gmail.com; 3 Department of Ophthalmology, Kasturba Medical College Manipal, Manipal Academy of Higher Education, Manipal 576104, India; sulatha.bhandary@manipal.edu; 4 Department of Computer Science, Design and Journalism, Creighton University, Omaha, NE 68187, USA

**Keywords:** diabetic macular edema, vision transformers, optical coherence tomography, deep learning, optimizers, disease, health, diabetes

## Abstract

**Background/Objectives:** Disorganization of the retinal inner layers (DRIL) is an important biomarker of diabetic macular edema (DME) that has a very strong association with visual acuity (VA) in patients. But the unavailability of annotated training data from experts severely limits the adaptability of models pretrained on real-world images owing to significant variations in the domain, posing two primary challenges for the design of efficient computerized DRIL detection methods. **Methods:** In an attempt to address these challenges, we propose a novel, self-supervision-based learning framework that employs a huge unlabeled optical coherence tomography (OCT) dataset to learn and detect clinically applicable interpretations before fine-tuning with a small proprietary dataset of annotated OCT images. In this research, we introduce a spatial Bootstrap Your Own Latent (BYOL) with a hybrid spatial aware loss function aimed to capture anatomical representations from unlabeled OCT dataset of 108,309 images that cover various retinal abnormalities, and then adapt the learned interpretations for DRIL classification employing 823 annotated OCT images. **Results:** With an accuracy of 99.39%, the proposed two-stage approach substantially exceeds the direct transfer learning models pretrained on ImageNet. **Conclusions:** The findings demonstrate the efficacy of domain-specific self-supervised learning for rare retinal pathological detection tasks with limited annotated data.

## 1. Introduction

Diabetic retinopathy is a major complication of diabetes mellitus, which is a chronic condition that affects a large population because of its widespread incidence. Diabetic macular edema (DME) remains the major cause of vision loss in diabetic individuals. According to the statistics by the International Diabetes Federation (IDF) Diabetes Atlas (11th edition), 1 among 9 individuals within an age group of 20–79 possesses diabetes, and 40% of the population are not aware of the fact that they have acquired the illness. IDF forecasts that 853 million people, or one of eight aged individuals, are expected to acquire the disease, indicating a rise of 46% in incidence by 2050 [1]. The diagnosing, tracking, and treatment of DR and DME have become vital in recent years. Imaging modalities like OCT provide high-resolution imaging of the retinal tissue, which allows for clear visualization of changes in the morphology of the retina in a non-invasive manner [2]. These imaging methods have become vital for guiding therapy and predicting patient outcomes, along with improved efficiency in diagnosis.

**Figure 1:**
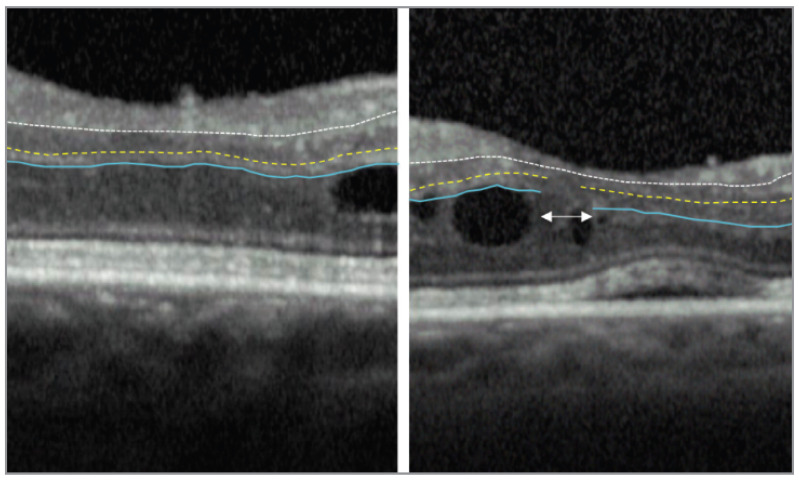
The figure at the left shows no DRIL highlighting the central 1000 μm foveal region of the OCT image. The ganglion cell layer-inner plexiform layer-inner nuclear layer (GCL-IPL-INL) interface is marked by the white broken line, while the INL-outer plexiform layer (INL-ONL) and OPL-outer nuclear layer (OPL-ONL) interfaces are highlighted by the yellow dashed and blue solid lines, respectively. The INL-OPL and OPL-ONL boundaries cannot be differentiated in the DRIL area indicated by the white double arrows in the right panel [4].

The retinal health and the structural abnormalities can be assessed with the help of noninvasive OCT imaging biomarkers. Subretinal fluid (SRF), cystic spaces within the intraretinal region, and DRIL, along with the thickness of the retina, are all crucial biomarkers on OCT that are designated as important identifiers of the disease severity, response to the treatment, and development. A vital imaging biomarker for DR, primarily DME, is DRIL apparent in OCT images. According to Sun et al. DRIL is defined as ”Disorganization of the retinal inner layers was defined as the horizontal extent in microns for which any boundaries between the ganglion cell–inner plexiform layer complex, inner nuclear layer, and the outer plexiform layer could not be identified” [3]. Figure 1 illustrates the absence and presence of DRIL in the OCT image.

The importance of DRIL as a reliable biomarker across multiple dimensions has been demonstrated by extensive clinical research. Novel possibilities have been offered by Deep Learning (DL) in the domain of healthcare imaging for identification of biomarkers and computer-based disease categorization. DL architectures have provided exceptional outcomes in the case of retinal image classification tasks, typically comparable to or even surpassing the results provided by doctors in the identification of a variety of OCT characteristics, including hyperreflective retinal foci (HRF), SRF, and structural differences in the ellipsoid zone (EZ).

Even though the current treatment methods for DME and DR are more successful, early detection of disease and treatment care are always beneficial. Use of automated disease identification tools that can categorize the patients’ groups to refer them to a suitable ophthalmologist is very important. Individuals with DME are often tested and scanned with the tool OCT in routine clinical practice. The OCT biomarker DRIL is proved to possess an association with RNFL layer thickening, decreased VA, EZ disruption, and retinal function impairment. It is very difficult to detect DRIL, as OCT variations are subtle, especially during the earlier manifestations of DME and DR [5]. Latest developments in the field of deep learning and artificial intelligence have shown enhanced potential for faster, computerized, and accurate health evaluation in the ophthalmic domain.

Unique and potential OCT biomarkers like DRIL are crucial, as there are no alternative methods in the literature to understand when people with DME may lose or gain their eyesight over time. Potential biomarkers like DRIL, the site of presence of fluid accumulation, and the variations in the morphology of the retina can all be identified with the help of OCT. To categorize people based on disease prognosis in research related to retinal health, identifying DRIL at baseline becomes important. Grading of DRIL by human experts is tedious, whereas automated methods can speed up the work and will allow for more extensive research to identify the connection within the DRIL development and response to treatments. A few researchers have attempted to use OCT with DL to diagnose ME associated with other disorders by leveraging the power of AI models. The method used in the implementation, the uniqueness of DRIL, and the idea of integrating DRIL detection with computer-based diagnostic methods are detailed in this research.

## 2. Related Work

This section details the state-of-the-art methods on DRIL detection and also the studies that define the association of DRIL with other OCT biomarkers. Sun et al. [3] defined DRIL as a novel biomarker where the study proved that extended severity of DRIL at baseline is correlated with decreased VA. Importantly, changes in DRIL over a time of initial four months were a crucial predictor of VA results over a period of eight months compared to central subfield thickness (CST). This study gave useful insights into the characteristics of DRIL and its associations, proving that DRIL is a noninvasive, reliable predictor of vision. Radwan et al. [6] revealed that in the presence of DME, the alterations in the resolution of DRIL are strongly correlated with future VA. In the case of severe DRIL, VA outcomes were very bad, but as DRIL cleared, there was improvement in the VA. This research proved that in the context of diabetic abnormalities, DRIL becomes a unique predictive biomarker. An extended follow-up study by Sun et al. [7] was focused on assessing 80 eyes with resolved and ongoing DME. They showed that, in both cases, DRIL spanning more than 50% of the foveal 1 mm region was always linked to lower VA. Grewal and Jaffe [4] defined DRIL as the “inability to distinguish between the outer plexiform layer, inner nuclear layer, and ganglion cell layer-inner plexiform layer complex.” They proved DRIL to be a reliable predictive biomarker for evaluating VA in cases of both uveitic and diabetic ME. They stated DRIL to be a crucial tool when it comes to counselling of patients and therapy. Various studies have documented substantially regarding the use of DRIL as a DME biomarker. Acon and Wu [8] report that there is still a need for discovering more about reliable biomarkers like DRIL, and people can use machine learning to plan treatment options and diagnoses that are built around various imaging modalities, even though OCT offers the best ways to manage DME.

Das et al. [9] analyzed the association of DRIL with the integrity of the outer retinal region. Using SD-OCT, they proved that a larger horizontal extent of DRIL is significantly associated with misalignment of EZ and the external limiting membrane (ELM), and both these factors contributed to lower best corrected VA (BCVA). They proved that DRIL being a surrogate marker of VA is also a predictor for increased morphological degeneration in the outer retina. These findings support the fact that DRIL acts as a pathophysiological link between inner and outer retinal disruption. Joltikov et al. [10] demonstrated a correlation of DRIL with decreased VA in individuals with initial stages of diabetic retinopathy (DR) but do not have ME. They also stated the therapeutic significance of DRIL and reported the possibility that DRIL seems to be an initial cellular consequence of diabetes. Nakano et al. [11] highlighted the role and impact of DRIL as an important tool for detailed visual functioning. They also showed that irrespective of the status of ME and VA, DRIL strongly correlates with the level of metamorphopsia in individuals with DME. These findings were supported by Nadri et al. [12], where they proved the association of DRIL with thinning of the retinal nerve fiber layer and EZ disruption. Di-Luciano et al. [13] performed a semantic assessment of seven research publications evaluating DRIL as an important biomarker of DME.

Most of these articles in the review employed SD-OCT scans with retrospective data-based cohort and cross-sectional designs. Throughout the findings provided by these reports, DRIL resolution was linked to improvement of vision, and the presence and increase in the extent of DRIL were constantly linked to decreased VA. Recent technical improvements in the field of DL have opened up new possibilities for automated DRIL detection. Singh et al. [5] used OCT images to categorize DRIL. This research also outlined the advantage of using DL in therapeutic healthcare decisions by designing the initial DL model for OCT biomarker categorization. Singh et al. [14] developed a DL-based convolutional neural network (CNN) achieving an accuracy of 88.3% in DRIL classification. However, this research lacked explainability even if it was one of the initial attempts to classify DRIL. A system based on fuzzy-logic design is employed by Tripathi et al. [15], leveraging DRIL, HRF, and cystoid spaces for determining DME severity from OCT scans. The method aimed at acquiring and reporting quantitative information to define classes of DME severity. Table 1 provides the details of various datasets used and the results obtained for the DRIL classification by the previous studies.

To bridge the gap between computer-based decision support systems and the clinical decision-making strategies, this work showed that rule-based methods that are easily interpretable, including different biomarkers such as DRIL for computerized assessment of DME severity, are achievable. In patients with DME, to identify hard exudates (HE) and to classify DRIL, Toto et al. [17] offered a DL-based system. They report that DL-supported DRIL detection is viable and provided one of the early extensive AI-based methods to combine classification pipelines with object detection-based strategies for DME biomarker assessment. Irrespective of the treatments given to patients, multiple studies have related the extent of DRIL to both VA at baseline and long-term results, as found by the extensive literature search that assesses DRIL-related articles by Tripathi et al. [19]. They reported the need for additional research to improve DRIL identification and use the results for providing individual patient care. Singuri et al. [16] demonstrate the fact that regardless of the subjective nature of DRIL, it has substantial association with VA status and DR. Ruiz-Medrano et al. [18] identified DRIL as a vital biomarker of DME on OCT by performing a study to predict treatment options. It is proved that DRIL presence, along with other biomarkers, leads to the failure of anti-VEGF treatments, necessitating the employment of additional treatment approaches.

## 3. Materials and Methods

**Figure 2:**
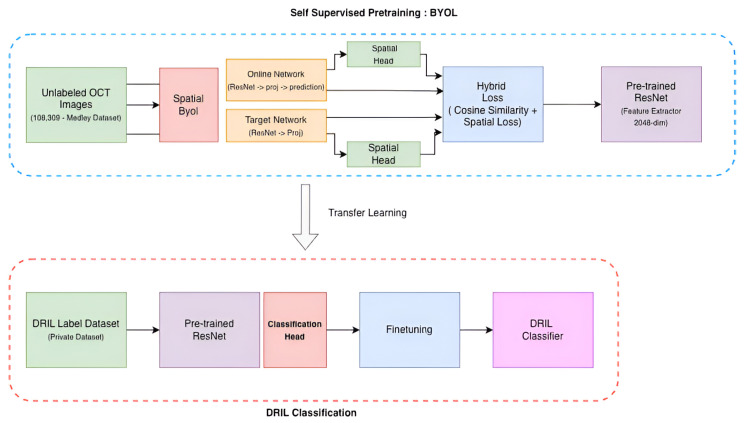
BYOL-based self-supervised training pipeline to learn pathologies in low data regime.

This section provides details of the method used, the datasets, the training pipeline, and Figure 2, utilized for classification of DRIL with limited annotated data conditions. We employ a Bootstrap Your Own Latent (BYOL) [20] learning framework based on self-supervision where a ResNet-50 backbone pretrained on ImageNet functions as an encoder. 108,309 unlabeled OCT images are used to refine this encoder to learn domain-related features of the retina in a class-agnostic approach. DRIL classification is thus achieved through the adaptation of this pretrained ResNet-50 encoder utilizing only 823 labeled images through transfer learning.

### 3.1. Mendeley Dataset

A large labelled OCT and chest X-ray collection dataset made available by Kermany et al. [21] is utilized for the research. 109,309 OCT images belonging to four categories—DME, Drusen, Normal, and Choroidal Neovascularization (CNV)—are contained in the dataset. All these retinal OCT images were obtained from the dataset of retinal scans performed at UC San Diego Health and the Shiley Eye Institute. The dataset is subdivided into training, validation, and testing groups to facilitate learning with self-supervision. Since this dataset is publicly available, diverse, and comprehensive for evaluating DL models in retinal image categorization, this dataset has become a common benchmark.

### 3.2. KMC Dataset

The private, labeled dataset was obtained from Kasturba Medical College (KMC), Manipal, MAHE, Manipal, from the Department of Ophthalmology. This dataset contains fovea-centered, anonymized, original OCT B scans that were used for evaluating the DRIL classification model. We have obtained ethical clearance for the dataset that was used from the Kasturba Medical College and Kasturba Hospital Institutional Ethics Committee, having assigned it an approval code IEC1-287/2022. The retrospective dataset contained horizontal, high-quality, fovea-centered B-scans having a signal strength greater than 7. The dataset was collected over a time period from January 2019 to August 2022. All these OCT images were captured using a certified Zeiss Cirrus HD OCT 5000 imaging device. The dataset contains 429 OCT images with the presence of DRIL, and 394 images do not have DRIL. The method used in the research uses expert-validated consensus annotations created by two experienced ophthalmologists from KMC Manipal. These ophthalmologists have immense professional experience treating patients with a multitude of eye disorders, including DR and DME, over a period of more than twenty-three years. The labelling of the images was done, and final reconciliations were performed to resolve discrepancies using a consensus protocol by these doctors. Thus, the dataset involved is highly reliable with clinical accuracy and relevance, and this provides a strong basis for the training and validation of DL models, preventing bias and promoting model generalization.

#### Inter-Observer Agreement Statistics

**Figure 3:**
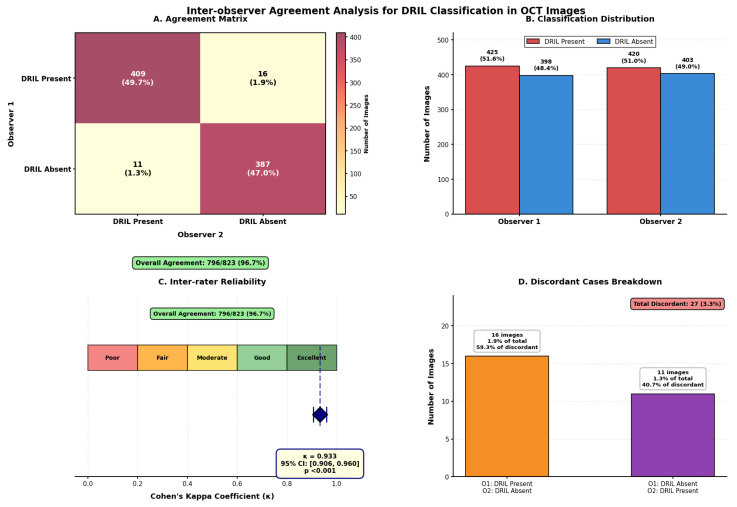
Panel (**A**) shows confusion matrix, Panel (**B**) shows agreement distribution, Panel (**C**) Cohen’s κ and Panel (**D**) shows the disagrement pattern.

Inter-observer variability metrics help us understand the consistency and differences in the annotations/labeling among the observers/doctors. Two experienced doctors, both having more than 23 years of clinical practice experience, have labelled the 823 OCT images for the presence and absence of DRIL. The metric known as Cohen’s kappa coefficient (κ), which considers the agreement between two sets of annotations provided by the doctors, is used to measure inter-observer variability. Among the 823 OCT scans, 796 (96.7%) scans were annotated with 100% agreement between both the doctors. Out of these 796 scans, both doctors agreed upon the presence of DRIL in 409 (49.7%) scans, and 387 (47.0%) were marked as DRIL-absent scans. But for the remaining 27 scans, discordant classifications were provided where 16 images (1.9%) were labelled as DRIL-present by Observer 1 and DRIL-absent by Observer 2. Also 11 images (1.3%) were annotated as DRIL-absent by Observer 1 and DRIL-present by Observer 2. Statistical assessment revealed satisfactory inter-observer agreement with Cohen’s κ = 0.933 (95% CI: 0.906–0.960, *p* < 0.001), which is really much more than the good reliability threshold (κ > 0.81). All 27 discordant cases were discussed together by both doctors in order to resolve the discrepancies and provide the final annotations through consensus labelling. The detailed inter-observer agreement analysis is shown in Figure 3. With Cohen’s κ = 0.933 (95% CI: 0.906–0.960, *p* < 0.001), the confusion matrix (Panel A) shows strong concordance, indicating excellent agreement beyond chance. There was 96.7% overall agreement (796/823 pictures). Observer 1 was classified as DRIL-present in 16 cases (59.3% of conflicts), Observer 2 was classified as DRIL-absent in 11 cases (40.7% of disagreements), and there were very few discordant cases (27 cases, 3.3%). As can be seen from Panel C’s kappa scale, our result is firmly in the “Excellent” area.

### 3.3. Reprsentation Learning in Anamtomical Context

Standard BYOL applies global average pooling to the encoder’s final feature map to obtain a single global representation of the image. This pooled vector is then passed through a projection MLP and a prediction MLP. While this approach works well for natural image representation learning, it discards all spatial structure. In medical imaging, specifically for OCT, spatial information is essential, as the pathologies are localized and retinal layers follow a fixed geometric structure. To address this, we extend BYOL with spatially aware feature learning and introduce a spatial self-supervision loss aimed at preserving structural information represented with Equation (1). Instead of relying solely on the deepest feature map, we extract latent representations at multiple spatial resolutions from the ResNet-50 backbone.

(1) $$\left\{s_{1},s_{2}\right\}=f_{\theta }\left(x\right),\;s_{i}\in R^{B\times C_{i}\times H_{i}\times W_{i}}$$

$$s_{1}\in R^{B\times 1024\times 14\times 14}$$

$$s_{2}\in R^{B\times 2048\times 7\times 7}$$

Here, $s_{1},s_{2}$ correspond to the feature maps at increasing network depth and decreasing spatial resolution. The final latent representation at $s_{2}$ is used for the adaptive average pooling as the standard BYOL implementation. This branch keeps the standard BYOL objective and maintains compatibility with the global downstream task. For spatial learning, we select $s_{1}$ as the input to our Spatial Branch. This layer provides an ideal balance between spatial granularity with 196 spatial locations and rich semantic content with 1024 channels. We process $s_{1}$ through our custom Spatial Projection Head and Spatial Prediction Head, both of which preserve the spatial grid. The Spatial Projection Head applies a 3×3 convolution followed by batch normalization and non-linearity, followed by a 1×1 convolution with batch normalization, producing a lower-dimensional spatial representation without destroying location information. The Spatial Prediction Head takes this spatial dimensional representation as input and applies two CNN blocks, yielding a representation, which plays the role of BYOL predictor for the spatial branch. This setup avoids collapsing the feature map into a single vector and instead learns a dense field of spatial predictions, allowing the model to retain anatomical structure.

To capture global semantics and anatomical structure, we introduce a hybrid loss that integrates the standard BYOL global objective with our spatial self-supervision objective. The global branch operates on the deepest feature map $s_{2}$. Considering $p_{1}^{g},p_{2}^{g}$ denote the global predictions for two augmented views, and $z_{1}^{g},z_{2}^{g}$ denote the corresponding target projections obtained from $s_{2}$, the global BYOL loss is given by the Equation (2).

(2) $$L_{\mathrm{global}}=\frac{1}{2}\left(\ell_{\cos}\left(p_{1}^{g},z_{2}^{g}\right)+\ell_{\cos}\left(p_{2}^{g},z_{1}^{g}\right)\right),\;\ell_{\cos}\left(a,b\right)=2-2\frac{a\cdot b}{∥a∥\;∥b∥}$$

In parallel, the spatial branch operates on $s_{1}$, preserving its H×W resolution. After passing $s_{1}$ through the Spatial Projection and Prediction Heads, we obtain spatial prediction maps $P_{1},P_{2}\in R^{B\times d_{s}\times H^{\prime }\times W^{\prime }}$ for the two augmented views. The target network produces corresponding spatial projections $Z_{1}^{\prime },Z_{2}^{\prime }$ with the same dimensions. We compute the cosine similarity at each location of the 14 × 14 location for the 128-dimensional vector and take the mean of the cosine similarity across all the locations, formally given by (3).

(3) $$L_{\mathrm{spatial}}=2-2\cdot E_{b,h,w}\left[\langle {\hat{P}}_{b,:,h,w},\;{\hat{Z}}_{b,:,h,w}^{\prime }\rangle \right],$$

and apply the same symmetric formulation:

$$L_{\mathrm{spatial}}=\frac{1}{2}\left(\ell_{\mathrm{sp}}\left(P_{1},Z_{2}^{\prime }\right)+\ell_{\mathrm{sp}}\left(P_{2},Z_{1}^{\prime }\right)\right)$$

Finally, we combine the global and spatial objectives into the hybrid loss:

$$L_{\mathrm{hybrid}}=\left(0.5\right)\;L_{\mathrm{global}}\;+\;\left(0.5\right)\;L_{\mathrm{spatial}}$$

This hybrid loss enables the model to learn global semantics through $s_{2}$ and local anatomical representations through the spatial supervision applied to $s_{1}$. The resulting encoder captures both high-level disease context and fine-grained retinal morphology properties that are critical for OCT lesion localization and segmentation downstream.

**Figure 4:**
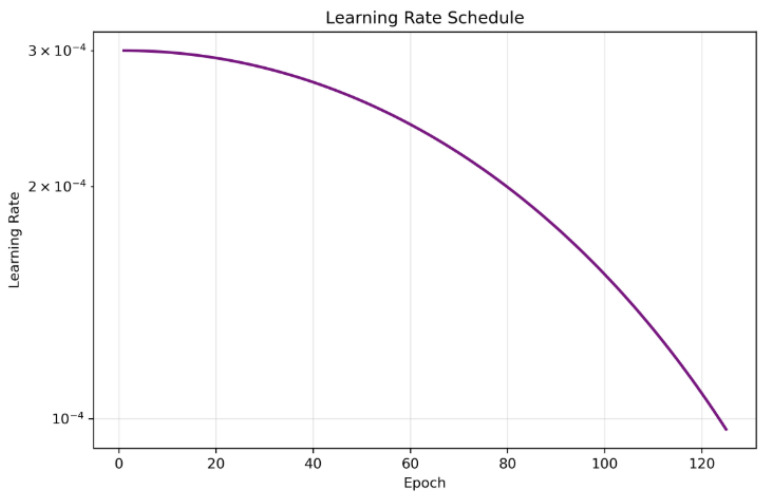
Figure showing the gradual decay of the learning rate over 125 epochs, enabling stable convergence of spatial BYOL.

**Figure 5:**
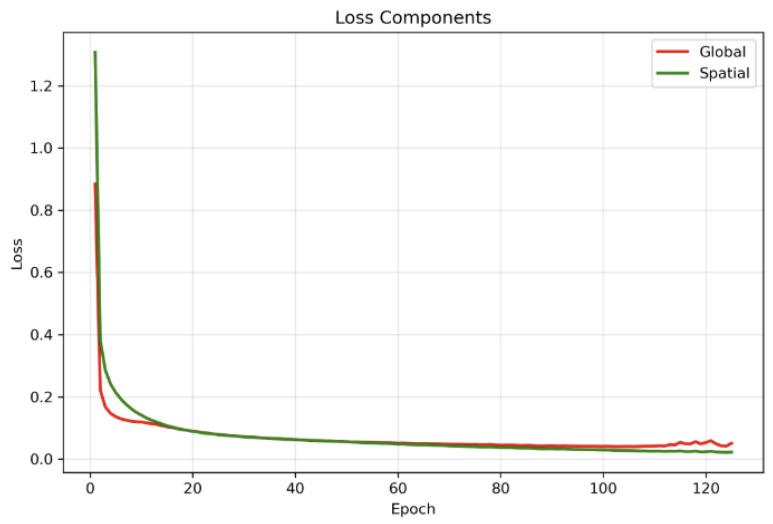
Curves indicating global loss vs. spatial loss during training.

**Figure 6:**
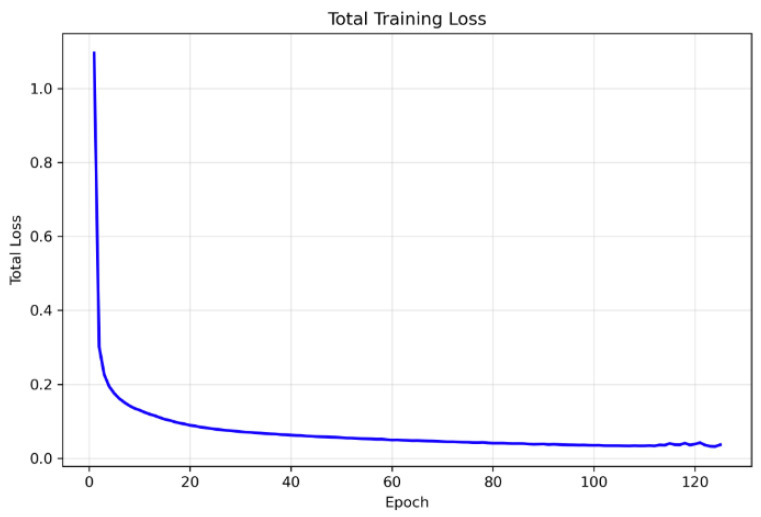
The complete training loss curve indicates stable representation learning.

We trained the spatial BYOL encoder for 125 epochs with early stopping conditions, with a patience of 25 epochs and a batch size of 64 and gradient accumulation over 4 consecutive mini-batches, yielding an effective batch size of 256 for each optimizer update. This configuration allowed us to maintain a large effective batch size. We used the AdamW optimizer with initial learning rate $3\times 10^{-4}$ and weight decay $1\times 10^{-4}$, together with a cosine annealing learning-rate schedule over the 100 epochs. We employed mixed-precision training and gradient clipping with max norm =1.0; all executions were carried out on NVIDIA A100 GPUs. The online and target encoders were coupled via an exponential moving average update with decay τ=0.996. The total training loss combined the global BYOL loss and the spatial BYOL loss with equal weighting ($\lambda_{\mathrm{spatial}}=0.5$), and the best checkpoint was observed at the 100th epoch and was selected as the backbone based on the minimum total BYOL loss on the training set, all of which are depicted in Figure 4, Figure 5 and Figure 6. Since the framework is fully self-supervised and does not use class labels, all four disease categories—CNV, DME, DRUSEN, and NORMAL—contribute equally to representation learning. This class-independent pretraining encourages the learned features to capture a broad range of OCT characteristics across disease types, improving the generalizability of the downstream models.

### 3.4. Finetuning for DRIL Identification

We employed the trained encoder for binary classification of DRIL after the self-supervised pretraining. The pretrained BYOL, along with a lightweight classification head, together made the classification model. Two fully connected layers with dropout regularization and batch normalization were included as a classification head. The classification head is responsible for application-specific prediction boundaries, and the high-level features are extracted by the encoder for detection of DRIL. Kaiming initialization was used for the linear layers of the classification head for random initialization.

In order to avoid catastrophic forgetting to enable application-specific adaptation, we employed a two-phase approach of fine-tuning that’s similar to pretraining. The classification head was made trainable, and the pretrained encoder was frozen. This enables the classifier that’s randomly initialized to adapt and learn patterns and features utilizing the pretrained characteristics without disturbing the learned representations. During this phase a very small quantity (2.6 M) of parameters were trainable while taking into consideration 73 M parameters. End-to-end fine-tuning is performed by unfreezing the encoder. The learning rate was lowered by 90% to allow for adaptation of the pretrained characteristics to characteristics that are specific to DRIL detection while promoting stability in learning. Fine-grained adaptation of both the high and low-level characteristics is allowed with this phase for optimized DRIL detection outcomes.

**Figure 7:**
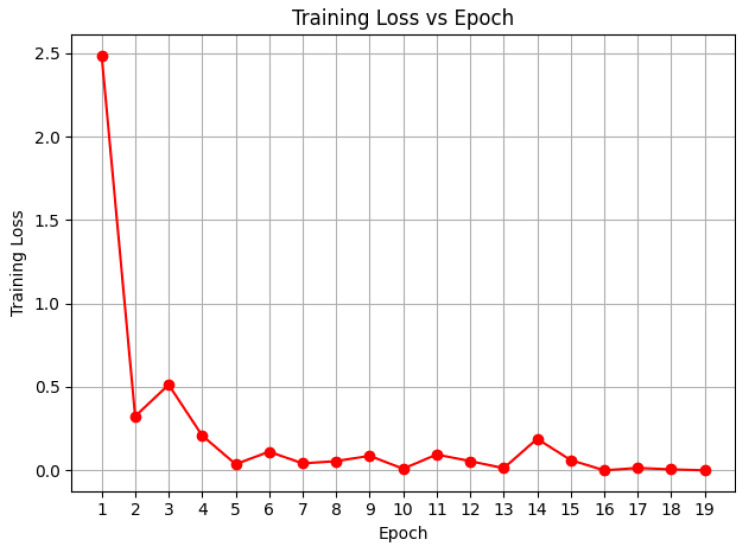
Finetuning Loss Curves for DRIL Classification.

**Figure 8:**
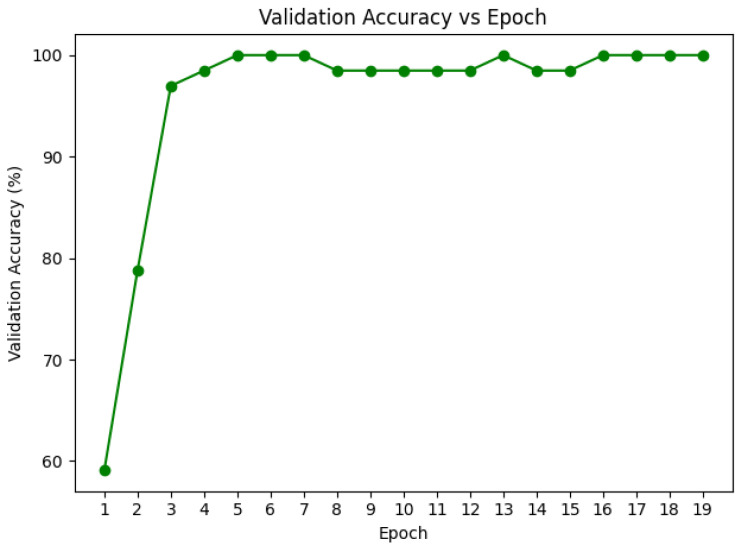
Validation Accuracy curves for DRIL fine-tuning. A 10% validation split (66 images) was held out from the training set to monitor generalization and prevent overfitting.

We utilized inverse frequency class weights with a loss function of weighted cross-entropy and the AdamW optimizer with a batch size of 32. When validation accuracy plateaued, dynamic reduction of learning rate was allowed with the ReduceLROnPlateau scheduler, thus enabling lighter optimization in the later training stages. To provide stability during training, gradient clipping was enabled with a norm of 1.0. The efficiency of the self-supervised method was demonstrated with the training converging within an average of 20–25 epochs observed in Figure 7 and Figure 8, because of the high-quality pretrained representations.

We have also made an attempt to evaluate the performance of the BYOL classifier with the other state-of-the-art CNN models in our quest to finalize a best possible model for classification of DRIL OCT images. Six CNN models that are pretrained on the ImageNet dataset, EfficientNetB3, ResNet50, InceptionResNetV2, MobileNetV2, DenseNet169, and VGG16, were fine-tuned to classify no-DRIL and DRIL OCT images. An almost identical training protocol has been used to train all six CNN models. The image size specifications were changed according to the requirements of the specific models to optimize the outcomes of the models. The parameters for which different values have been used are input image size and the type of augmentations used to get optimal results, which have been listed in Table 2. A stratified dataset split (80% for training, 10% of training data used for validation, and 20% for testing) has been used. Each model was evaluated for baseline performance with the backbone frozen and trained for 15 epochs, which was followed by complete fine-tuning of all the layers for another 15 epochs. All models were trained with the Adam optimizer and with a learning rate of $1\times 10^{-3}$ during the frozen stage and $1\times 10^{-4}$ during the fine-tuning phase. Across all architectures a batch size of 32 has been used with binary cross-entropy loss. The best model checkpoint obtained with minimum validation loss was saved for further evaluation. An independently held-out test set of 165 testing samples (20% of the entire dataset of 823 images) is used to assess the performance of all the fine-tuned models.

## 4. Results

Our experiments show that standard transfer learning baselines achieve strong performance on the DRIL classification task, with models such as ResNet50 and VGG16 reaching 98.18% accuracy. Table 3 provides a detailed comparison between our method and several pretrained state-of-the-art architectures. However, our proposed approach, Spatial BYOL with Hybrid loss, is able to outperform all baselines, achieving 99.39% accuracy with only a single misclassification on the test set.

Table 4 further contextualizes this improvement by comparing model sizes and convergence behavior. With 73 million parameters, of which only 27 million are updated during fine-tuning, our model performs better than the VGG-16 with over 138 million parameters and converges faster. Together, these results demonstrate that the proposed Spatial BYOL learns more expressive representations than existing pretrained models, enabling both faster convergence and improved final performance with substantially fewer trainable parameters.

**Figure 9:**
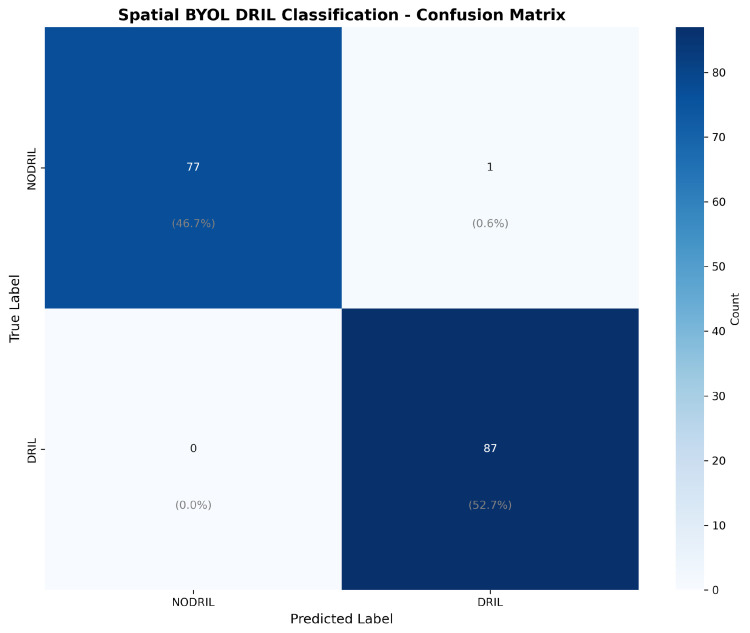
Confusion Matrix for the proposed spatial BYOL.

**Figure 10:**
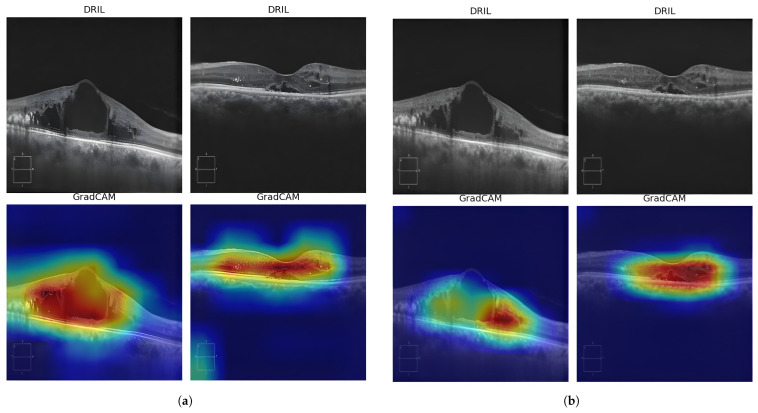

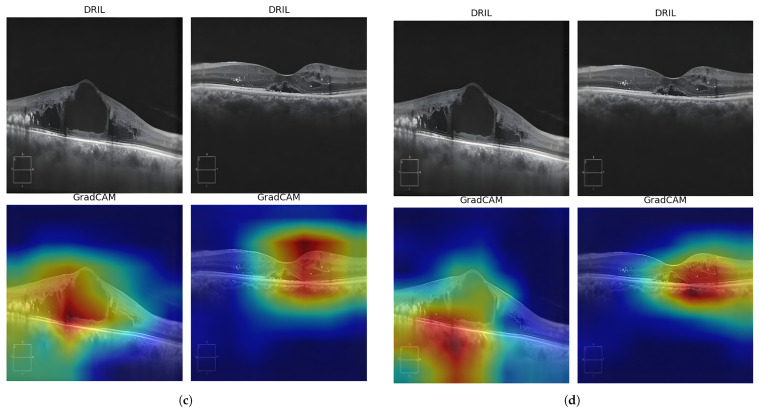
Grad-CAM heatmaps obtained for various models: (**a**) VGG16 (**b**) EfficientNetB3 (**c**) MobileNetV2 (**d**) ReNet50.

**Figure 11:**
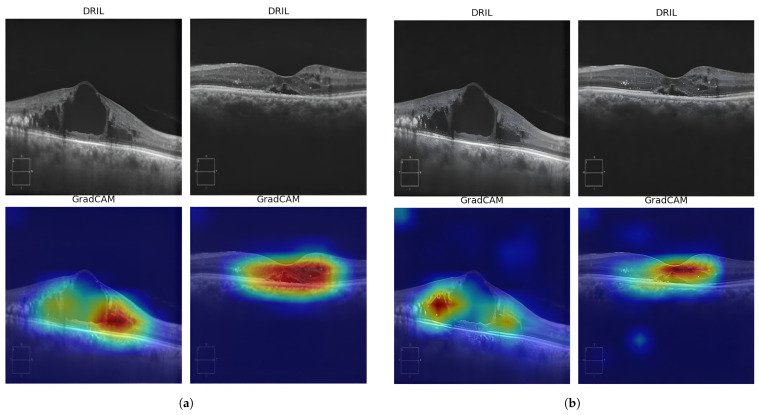

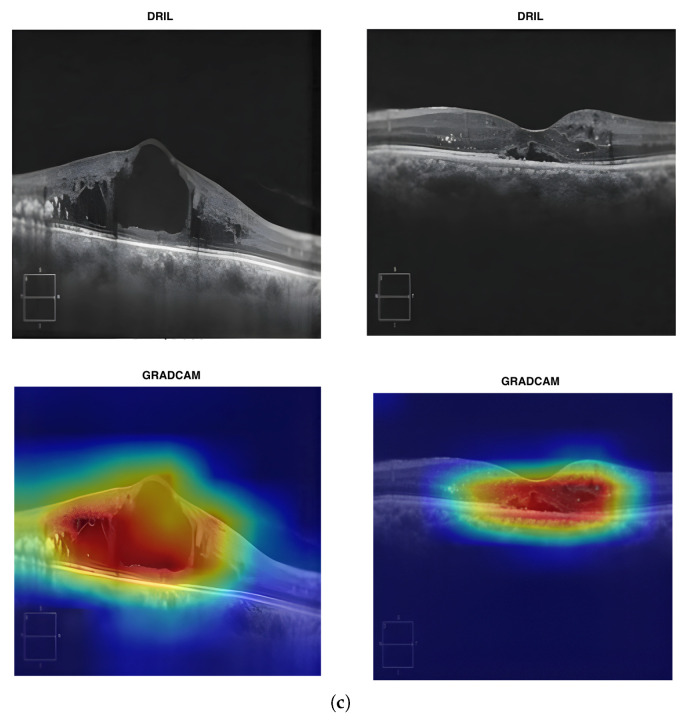
Grad-CAM heatmaps obtained for various models: (**a**) DenseNet169 (**b**) InceptionResNetV2 (**c**) Spatial BYOL.

The confusion matrix in Figure 9 reveals the model’s error pattern. Out of 78 No-DRIL cases, 77 were correctly classified, giving us a specificity of 98.72%. Our approach was able to identify all 86 DRIL cases correctly, yielding a 100% sensitivity with zero false negatives. This performance is particularly significant for clinical deployment, as failing to detect DRIL (false negatives) has greater clinical consequences than false alarms, which can be resolved through secondary review. Explainability becomes crucial for the AI model to be accepted clinically for DRIL detection. Gradient-weighted Class Activation Mapping (Grad-CAM) heatmaps provide useful information about which regions of the OCT images have contributed the most in making the DRIL prediction. Figure 10 and Figure 11 provide the Grad-CAM heatmaps obtained for various pretrained models along with the proposed spatial BYOL implementation.

## 5. Discussion

DRIL remains a difficult target for reliable detection in a clinical setting. In contrast to more overt retinal pathology such as large hemorrhages or exudates, DRIL appears as very subtle irregularity within the inner retinal layers, and often requires careful, targeted inspection for even experienced graders to confidently identify. These biomarkers and their associated structural changes alter the scan appearance only slightly, but still have clinical relevance, which makes algorithmic detection challenging. The problem is further amplified by the limited availability of high quality expert-annotated DRIL cases.

Supervised deep neural networks typically require large numbers of labeled examples to learn fine-grained and discriminative visual cues of this type. However, obtaining reliable labels for DRIL is costly, labor intensive, and slow, and this limits the achievable dataset scale in practice. Conventional transfer learning from natural image benchmarks also provides only marginal benefit because of the substantial domain mismatch. Feature extractors trained on everyday photographs tend to emphasize cues that are characteristic of object-centric scenes, such as sharp object boundaries, natural color statistics, and common surface textures. These learned inductive biases do not align well with retinal OCT, which is defined by layered biological structure, speckle characteristics, and subtle disease-related architectural changes.

To address the challenges of subtle pathological features, limited annotations, and domain shift, we propose leveraging self-supervised learning on large dataset of unlabeled OCT data. Our approach helps our model learn domain specific representations before being finetuned for pathology specific tasks. This class agnostic pretraining method allows the model to grasp basic OCT image characteristics, like retinal layer structures, tissue reflectivity patterns, speckle noise features, and anatomical spatial relationships without needing pathology labels. By training on diverse pathological conditions in an unsupervised manner, the model learns to encode retinal layer boundaries, variations in tissue texture, and structural organizations that are vital for generalizability. In comparison to supervised pretraining on a single pathology which would skew the learned features toward task-specific patterns and limit their transferability.

For OCT B-scans and for medical imaging, contrastive methods seem to be less well-suited. We hypothesize that this could be due to individual scans sharing highly similar global retinal structure, and disease-related changes often manifest as subtle, localized variations. This makes it difficult to construct truly “negative” examples. Images from different patients or even different disease categories can still be very similar at the global level, increasing the risk of false negatives and degrading representation quality. At the same time, our goal is to operate in relatively low-data and moderate-compute regimes, where transformer-based SSL approaches and masked-image-modeling variants, including DINO with VIT backbones, are typically data and computation intensive and therefore harder to deploy at full scale. We observe in our study that DINO with CNN-based backbones don’t perform equally well. In this context, self-distillation methods and BYOL offer a promising solution. BYOL does not rely on negative sampling, is compatible with convolutional backbones that naturally capture local retinal structure, and has been shown to perform well with smaller batch sizes and limited data. These properties make BYOL a natural choice for learning OCT-specific representations that remain sensitive to subtle pathology while respecting our computational constraints.

Our results establish self-supervised learning on domain-specific data as a viable strategy for creating foundation models in medical imaging. The pretrained BYOL encoder acts as a general feature extractor for OCT images. Capable of rapid adaptation for downstream tasks with minimal labelled data. The foundational model approach for specific imaging areas holds particular promise for rare pathologies and new biomarkers, where expert annotations are often limited. A single self-supervised pretraining phase on varied unlabeled OCT data can support multiple downstream applications, such as DRIL detection, drusen quantification, CNV classification, and layer segmentation through lightweight, task-specific heads. This approach amortizes computational cost of representation learning across various clinical applications.

## 6. Conclusions

We demonstrate a framework supported with self-supervised learning to address the critical issue of computerized DRIL detection with limited labeled data. Our research shows that domain-specific learning based on self-supervision can help improve over traditional transfer learning strategies, improved results and faster convergence with limited supervision show the ability of this approach to have potential to adapt to complex and rare pathologies where the labelled data is scarce. We acknowledge that testing on a limited dataset size may constrain the generalizability of our findings. Our future research focuses on exploring the adaptation of proposed self-supervised OCT pretraining to various other retinal imaging biomarkers and lesions. Further, investigation of self-supervised learning with other imaging modalities like fundus and OCT angiography incorporating the identification of different retinal disorders may be explored to move towards foundational models in ophthalmic diagnosis.

## Figures and Tables

**Table 1:** Summary of related studies on DRIL classification using OCT images.

Reference	Dataset/No. Images	Accuracy	Sensitivity	Specificity	Cohen’s κ	AUC	MCC
Sun et al. [3]	120 eyes	NR	NR	NR	NR	NR	NR
Radwan et al. [6]	70 eyes (43 DME, 27 non-DME)	NR	NR	NR	κ = 0.88–1.00	NR	NR
Sun et al. [7]	80 eyes	NR	NR	NR	κ = 0.69–0.77	NR	NR
Acón & Wu [8]	Review study	NR	NR	NR	NR	NR	NR
Das et al. [9]	102 eyes (80 patients)	NR	NR	NR	κ = 0.6–1.0	NR	NR
Joltikov et al. [10]	57 diabetic, 18 controls	NR	NR	NR	r = 0.98	NR	NR
Nakano et al. [11]	37 eyes	NR	NR	NR	CC = 0.93–0.99	NR	NR
Nadri et al. [12]	104 subjects (78 diabetic)	NR	NR	NR	κ = 0.85	NR	NR
Di-Luciano et al. [13]	7 studies (systematic review)	NR	NR	NR	NR	NR	NR
Singuri et al. [16]	2083 eyes (1175 patients)	NR	NR	NR	κ = 0.88	NR	NR
Singh et al. [5]	2392 images (417 eyes, 229 patients)	94.36%	NR	NR	NR	0.988	NR
Singh et al. [14]	5992 images (1201 eyes)	88.3%	82.9%	90.0%	κ > 0.85	NR	0.7
Tripathi et al. [15]	150 images	93.3%	NR	NR	NR	NR	NR
Toto et al. [17]	442 images	91.1%	91.1%	91.1%	κ = 0.82	91%	NR
Ruiz-Medrano et al. [18]	275 eyes (209 switch, 66 control)	NR	NR	NR	NR	NR	NR

NR: Not Reported in the original study.

**Table 2:** Input image sizes and the type of augmentations used for the CNN models.

Model	Input Size	Augmentations
DenseNet169	224 × 224	Resize; Random Horizontal Flip; Rotation (±10°); Color Jitter; ImageNet Normalization
EfficientNetB3	300 × 300	Resize; Random Horizontal Flip; Rotation (±20°); Color Jitter; Random Zoom; RandomResizedCrop; ImageNet Normalization
ResNet50	224 × 224	Resize; Random Horizontal Flip; Rotation (±15°); Color Jitter; RandomAffine; ImageNet Normalization
VGG16	224 × 224	Resize; Random Horizontal Flip; Rotation (±10°); Color Jitter; ImageNet Normalization
MobileNetV2	224 × 224	Resize; Random Horizontal Flip; Rotation (±10°); Color Jitter; ImageNet Normalization
InceptionResNetV2	299 × 299	Resize; Random Horizontal Flip; Rotation (±10°); Color Jitter; Normalization (mean = 0.5, std = 0.5)

**Table 3:** Comparison of DRIL Classification Performance Across Different Approaches.

Author/Approach	Accuracy (%)	Precision (%)	Recall (%)	F1-Score (%)
Pretrained VGG16 + Finetuning	98.18%	96.63%	100.00%	98.29%
Pretrained EfficientNetB3 + Finetuning	96.96%	96.55%	97.67%	97.10%
Pretrained MobileNetV2 + Finetuning	97.57%	96.59%	98.83%	97.70%
Pretrained ResNet50 + Finetuning	98.18%	96.62%	100.00%	98.28%
Pretrained DenseNet169 + Finetuning	97.57%	96.59%	98.83%	97.70%
Pretrainied InceptionResNetV2 + Finetuning	96.96%	96.55%	97.67%	97.10%
Pretrained VIT-16 + Finetuning	98.18%	98.19%	98.18%	98.18%
Pretrained VIT-32 + Finetuning	96.36%	96.62%	96.36%	96.37%
Moco Backbone + CNN Finetune Head	98.79%	98.79%	98.79%	98.79%
SimCLR Bacbone + CNN Finetune Head	95.76%	96.08%	95.76%	95.74%
Dino Bacbone + CNN Finetune Head	96.96%	96.55%	97.67%	97.10%
**BYOL + CNN Finetune Head**	98.79%	98.82%	98.79%	98.79%
**Proposed/Spatial BYOL + Finetune + Hybrid Spatial Aware Loss Fn**	**99.39%**	**99.40%**	**99.39%**	**99.39%**

**Table 4:** Model and respective sizes comparison with other SSL based approaches.

Model	Total Paramaeters	Convergence Epochs for DRIL Classification
VGG-16	138 Million	30
EfficientNetB3	12 Million	20
ResNet50	25 Million	22
DenseNet169	12 Million	18
InceptionResNetV2	55 Million	26
VIT-B 16	86 Million	18
Vit-B 32	88 Million	16
SimCLR	27 Million	28
Moco	55 Million	29
Dino	98 Million	33
BYOL	68 Million	22
Spatial BYOL	73 Million	19

## Data Availability

The public data presented in the study are openly available in the Mendeley database at [https://doi.org/10.17632/rscbjbr9sj.3]. The private dataset generated and analyzed during this study is not publicly available due to ethical considerations. However, they can be obtained from the corresponding author upon reasonable request. Code for implementation can be found here [https://github.com/Tulsani/Spatial-Byol] (accessed on 2 November 2025).
